# Supplementary material for: STAT3 Mediated miR-30a-5p Inhibition Enhances Proliferation and Inhibits Apoptosis in Colorectal Cancer Cells
Source: Int J Mol Sci. 2020 Oct 3;21(19):7315. doi: 10.3390/ijms21197315 (PMC7583989; doi:10.3390/ijms21197315)
Supplement: Supplementary file 1 [file ijms-21-07315-s001.zip › ijms-933574-sup/ijms-933574-sup-proof done.docx]

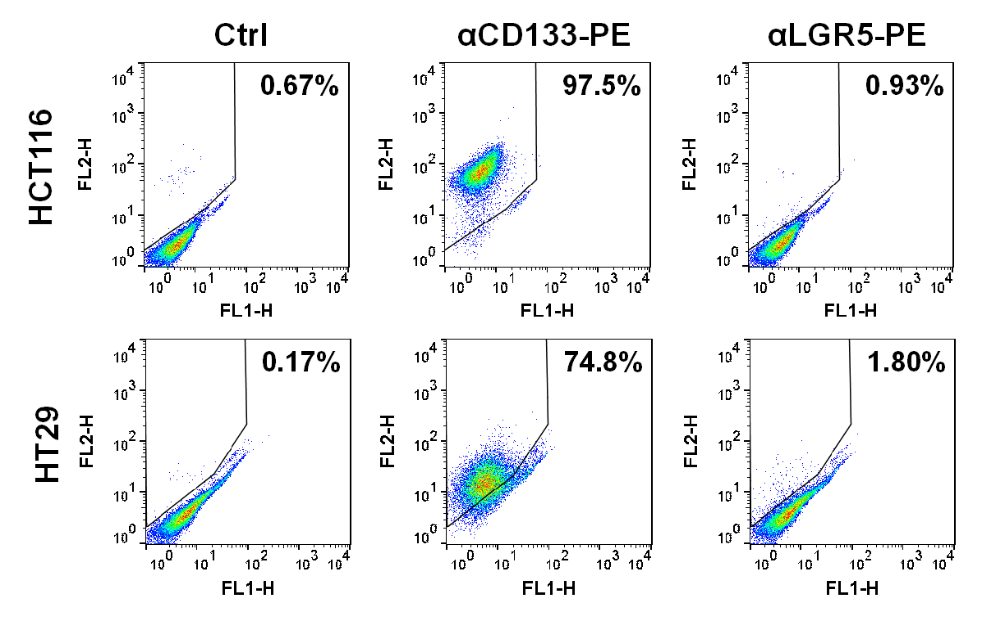


**Figure S1.** CD133 expressed in colorectal HCT116 and HT29 tumor cells**.** Flow cytometry revealed that both HCT116 and HT29 were CD133-positive and LGR5-negative. The cells were stained with anti-CD133-PE or anti-LGR5-PE for 30 min at room temperature. FL2-H presents PE signal.


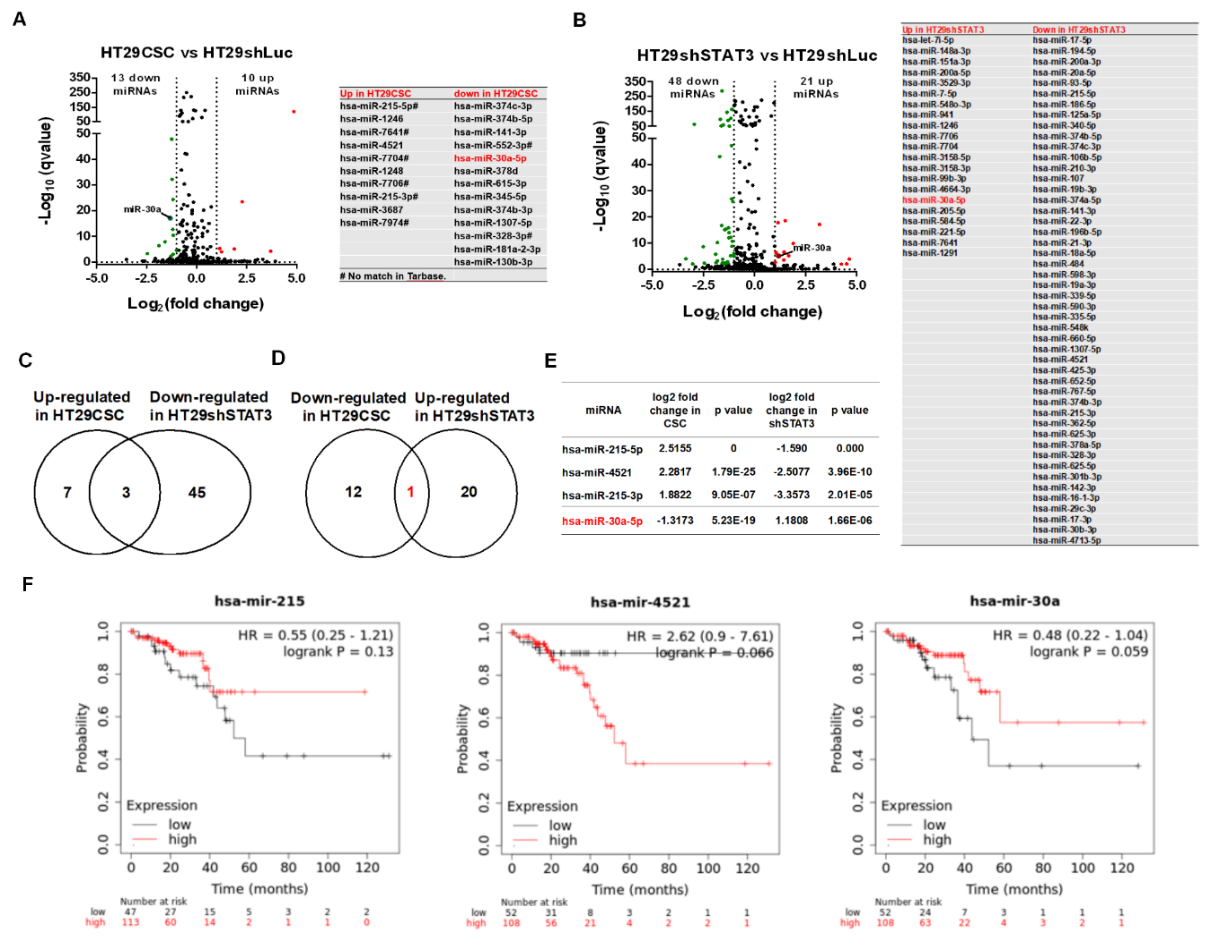


**Figure S2.** Kaplan–Meier plots (http://kmplot.com/analysis/) revealed that a high miR-4251 level reduced the probability of survival in patients with rectum adenocarcinoma (n = 160, *p* = 0.065); by contrast, the probability of survival increased following an increase in the miR-30a level (n = 160, *p* = 0.059).

**
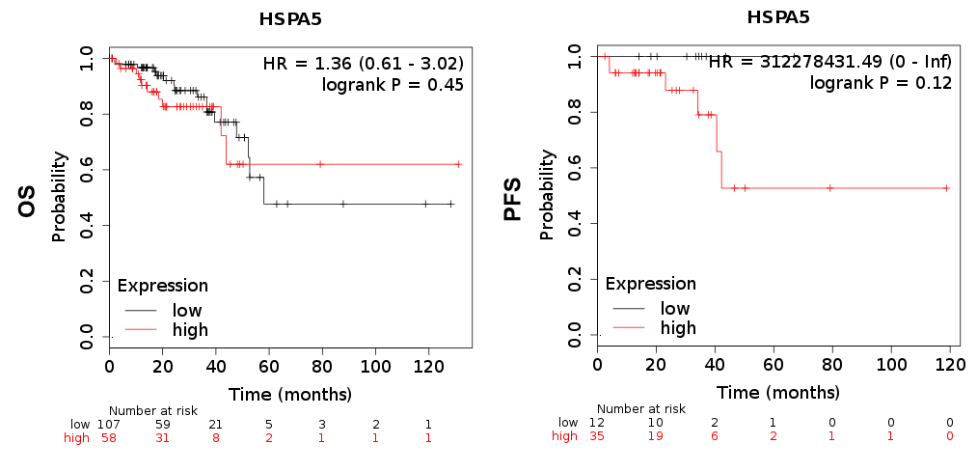
**

**Figure S3.** Kaplan–Meier plots revealed that a high HSPA5 level was associated with poor survival (progression-free survival, PFS) in patients with rectum adenocarcinoma (n = 165, *p* = 0.12).
